# Supportive Care Interventions for People With Cancer Assisted by Digital Technology: Systematic Review

**DOI:** 10.2196/24722

**Published:** 2021-10-29

**Authors:** Michael Marthick, Deborah McGregor, Jennifer Alison, Birinder Cheema, Haryana Dhillon, Tim Shaw

**Affiliations:** 1 Research in Implementation Science and eHealth Group Faculty of Medicine and Health University of Sydney Camperdown Australia; 2 Faculty of Science, Medicine and Health University of Wollongong Wollongong Australia; 3 Faculty of Medicine and Health University of Sydney Sydney Australia; 4 Sydney Local Health District Sydney Australia; 5 School of Health Sciences Western Sydney University Penrith Australia; 6 Centre for Medical Psychology & Evidence-based Decision-making School of Psychology University of Sydney Sydney Australia

**Keywords:** digital health, telehealth, eHealth, neoplasm, supportive care, systematic review, mobile phone

## Abstract

**Background:**

Although relatively new, digital health interventions are demonstrating rapid growth because of their ability to facilitate access and overcome issues of location, time, health status, and most recently, the impact of a major pandemic. With the increased uptake of digital technologies, digital health has the potential to improve the provision of supportive cancer care.

**Objective:**

This systematic review aims to evaluate digital health interventions for supportive cancer care.

**Methods:**

Published literature between 2000 and 2020 was systematically searched in MEDLINE, PubMed, Embase, PsycINFO, Cochrane Central Register of Controlled Trials, and Scopus. Eligible publications were randomized controlled trials of clinician-led digital health interventions to support adult cancer patients. The interventions included were determined by applying a digital health conceptual model. Studies were appraised for quality using the revised Cochrane risk of bias tool.

**Results:**

Twenty randomized controlled trials met the inclusion criteria for the analysis. Interventions varied by duration, frequency, degree of technology use, and applied outcome measures. Interventions targeting a single tumor stream, predominantly breast cancer, and studies involving the implementation of remote symptom monitoring have dominated the results. In most studies, digital intervention resulted in significant positive outcomes in patient-reported symptoms, levels of fatigue and pain, health-related quality of life, functional capacity, and depression levels compared with the control.

**Conclusions:**

Digital health interventions are helpful and effective for supportive care of patients with cancer. There is a need for high-quality research. Future endeavors could focus on the use of valid, standardized outcome measures, maintenance of methodological rigor, and strategies to improve patient and health professional engagement in the design and delivery of supportive digital health interventions.

**Trial Registration:**

PROSPERO CRD42020149730; https://www.crd.york.ac.uk/prospero/display_record.php?RecordID=149730

## Introduction

### Background

Approximately 18.1 million new cancer cases and 9.6 million cancer-related deaths occurred globally in 2018 [1]. The rising tide of cancer diagnoses in many developed countries has been attributed to both population aging and the increasing prevalence of primary risk factors, including physical inactivity, obesity, and metabolic disease [1,2]. As the cancer population continues to grow, there is an urgent need to improve supportive care services [2].

Supportive care focuses on assisting people with cancer and their families to cope with the disease and its treatment [3]. The management of cancer treatment-related symptoms and side effects and the maintenance of health-related quality of life from early diagnosis to end-of-life are key aims of supportive cancer care [3,4]. Supportive care interventions vary and may involve multidisciplinary team support, including doctors, nurses, pharmacists, and allied health professionals [5]. Recently, a shared follow-up approach between primary and secondary providers has been promoted to successfully meet increasing demands for survivorship care [6,7], as innovative methods for long-term cancer care are constantly needed [2].

There have been ongoing attempts to improve access to supportive care cancer services through the use of digital health technology [8]. Digital health interventions, with telemedicine as its oldest form dating back to the 1920s, have been increasing dramatically in recent years [9]. The terms *digital health* and *eHealth* are frequently used interchangeably, with numerous varied definitions. Eysenbach [10] defined eHealth as *an emerging field in the intersection of medical informatics, public health, and business, referring to health services and information delivered or enhanced through the internet and related technologies*. Elbert [11] and McLean et al [12] assert 3 key elements of digital health: (1) data obtained from the patient, (2) electronic transfer of data over a distance, and (3) patient-tailored feedback from a health care professional. Furthermore, a recent conceptual model proposed by Shaw et al [13] acknowledges the role of telehealth consultations, web-based forums, mobile devices and apps, and social media, in enabling real-time communication between health professionals and consumers.

Recent systematic reviews evaluating the impact of digital health interventions on health and health care costs provide promising evidence of effectiveness and cost-effectiveness [11,14]. Digital health has demonstrated potential in engaging people in their care [15], including as a tool for the treatment and self-management training of chronically ill patients [16,17]. Digital health interventions have been shown to be effective for managing cancer-related fatigue [18], may improve physical activity among cancer survivors [19], and can lead to positive effects addressing the supportive cancer care needs of individuals with different preferences and priorities [20]. Cancer survivors have been found to have a positive attitude toward digital health [21], suggesting that digital health interventions have the potential to overcome common challenges associated with access to supportive care in this population. Health professional-led, digital health–enabled, supportive care interventions may prove particularly useful in increasing accessibility of services to those with limited access because of location, health, time, and public health emergencies [22-30]. Despite the abundance of recent digital health literature, there remains an acknowledged lack of quality evidence regarding the effectiveness of supportive digital health care interventions for people with cancer [19,20,31-33].

### Objective

Over the past several decades, studies have investigated the implementation and effects of digital health interventions in people with cancer. In previous systematic reviews evaluated in 2013, the design features of supportive digital health interventions for patients with cancer [20]; in 2014, the use of technology in cancer follow-up [31]; in 2015 and 2017, the effect of telehealth interventions in cancer survivors’ general quality of life [32,34], and in 2020, the benefits and limits of digital health for optimal supportive care in oncology [35]. The last review by Aapro et al [35] conducted an article search up to November 2018 and focused on the technical features of digital technologies. This is a rapidly growing area of health care because of advances in information technology and the uptake of digital technologies by both health professionals and patients. Therefore, this systematic review aims to explore the effect of supportive care interventions assisted by digital technologies on the outcomes of patients with cancer.

## Methods

### Search Strategy

This systematic review was registered in the PROSPERO (International Prospective Register of Systematic Reviews) and PRISMA (Preferred Reporting Items for Systematic Reviews and Meta-Analyses) guidelines [36]. A search wa**s** performed in August 2020, using the following web-based databases: MEDLINE (OvidSP, Wolters Kluwer), PubMed (National Center for Biotechnology Information, US National Library of Medicine), Embase (OvidSP, Wolters Kluwer), PsycINFO (American Psychological Association), Cochrane Central Register of Controlled Trials (John Wiley & Sons), and Scopus (SciVerse, Elsevier). Three main keywords were searched: *supportive care*, *digital health*, and *cancer patients*. Additional search terms were included based on synonyms of these keywords and medical subject headings. Multimedia Appendix 1 indicates the search strategy used.

### Inclusion Criteria

The inclusion criteria were as follows: (1) studies in English describing a randomized controlled trial (RCT), published between January 2000 and August 2020; (2) intervention recipients were adults with a diagnosis of cancer; (3) involved clinician-led digital health interventions; and (4) interventions implemented to provide supportive cancer care.

The determination of digital health interventions was on the basis of the conceptual model of Shaw et al [13], which consists of 3 core domains:

1. Health in our hands: Using digital technologies to monitor, track, and inform health, for example, smartphones, tablets, clinical devices, mobile sensors and wearables, apps, social media, and web-based information.
2. Interacting for health: Using digital technologies to enable health communication among practitioners and between health professionals and clients or patients, for example, traditionally dominated by teleconferencing and videoconferencing, this domain increasingly includes a range of synchronous and asynchronous tools, such as SMS and push notifications from mobile apps, dedicated portals, social media platforms, and virtual or simulated therapy tools.
3. Data enabling health: Collecting, managing, and using digital health data, for example, technologies that provide expanded knowledge and insights about the health of an individual, community, or population.

To be included in the review, it was essential that the intervention satisfied the 2 domains *health in our hands* and *interacting for health*. The third domain, *data enabling health* was deemed nonessential because of the known inconsistent reporting of these criteria. The essential criterion *health in our hands* was captured in the *Intervention* column and outlines the nature of digital health experience. *Interacting for health* was captured in the *Interactions* column of review data and outlines the individuals involved in any form of communicative exchange that supports the health and well-being of the patient and caregiver.

Studies with interventions involving automated systems, such as interactive voice response and similar web-based systems, to monitor symptoms were included if the intervention featured an internet or web-based component and triggered health professional or researcher involvement when a threshold, such as a pain score, was reached.

### Exclusion Criteria

Studies were excluded if they were (1) not RCTs; (2) reported only self-managed interventions, patient-to-patient interventions, prevention tools, or alternative treatments, or (3) focused solely on interventions involving telephone delivery that replicated a clinical service.

### Data Extraction and Synthesis

Two reviewers (MM and DM) independently reviewed the titles and abstracts, followed by a full-text review of all publications. In cases of disagreement, a consensus was sought through discussion. Disagreement persisted for 4 studies; therefore, a third reviewer (TS) was consulted to adjudicate.

Endnote software (Clarivate Plc) [37] was used to manage references, and Covidence software (Veritas Health Innovation Ltd) was used to import and extract studies [38]. Two reviewers (MM and DM) independently applied the revised Cochrane risk of bias tool (The Cochrane Collaboration) [39] to establish the quality of the included studies. A matrix was developed by the authors and applied in the collection and analysis of structured data. Matrix criteria also included whether a study adhered to the CONSORT (Consolidated Standards of Reporting Trials) eHealth Checklist [40], a tool developed to improve the standard of reporting in digital health trials.

### Quality Assessment

Using the revised Cochrane Collaboration’s tool for assessing risk of bias (RoB) in randomized trials (Risk of Bias 2.0) [39], each study’s methodological quality was assessed in 5 major domains: randomization process, deviation from intentional interventions, missing outcomes, measurement of outcomes, and selection of reported results. The RoB for each domain was rated as *some concerns*, *low*, or *high*. The overall RoB for each study was rated as *some concerns* or *high*.

## Results

### Study Selection

**Figure 1:**
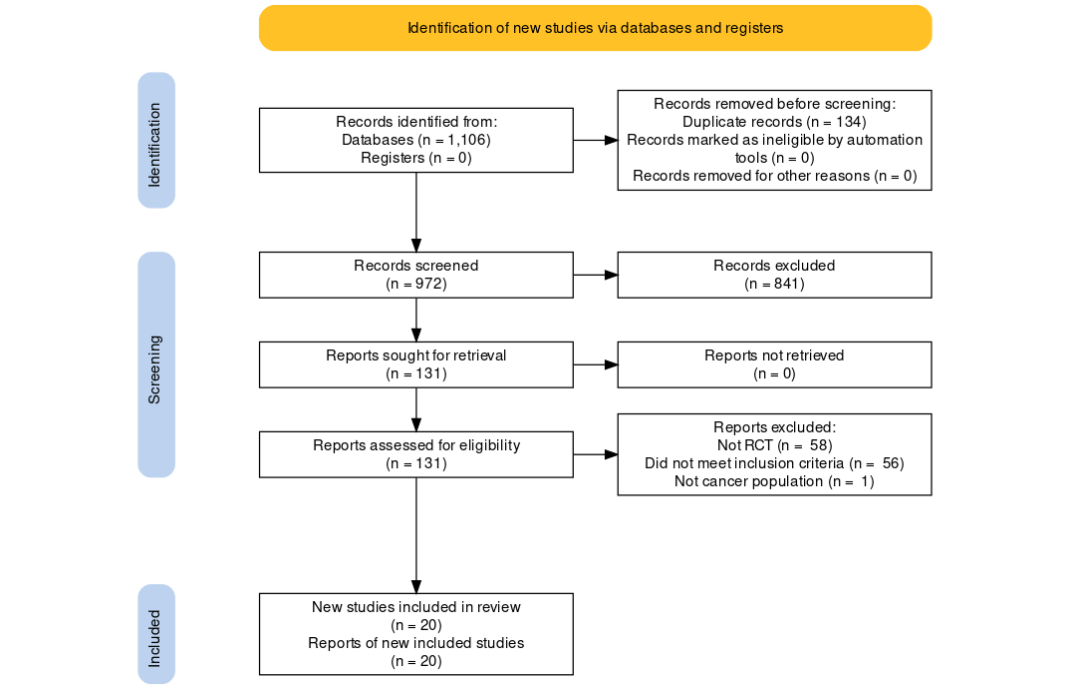
PRISMA (Preferred Reporting Items for Systematic Reviews and Meta-Analyses) flow diagram of the search and study selection process. RCT: randomized controlled trial.

The initial search generated 972 records. After title and abstract review, 135 publications were retained. The full text for each of these 135 publications was reviewed for eligibility, resulting in the identification of 17 publications. Three additional studies were found using a reference search, generating a total of 20 digital health–enabled supportive cancer care interventions for inclusion in the review (Figure 1).

### Study Characteristics

#### Population

Details of the study design and characteristics are given in Multimedia Appendix 2 [41-60]. Although the search was published in 2000, the earliest result identified was published in 2007, and the remaining included studies were published after 2009. A total of 20 studies were conducted across 8 countries [41-48], with 11 from the United States. The sample sizes ranged from 52 to 516 participants, with a median of 118.

Of the 20 included studies, 12 (60%) were designed for participants within a single tumor stream. Interventions for breast cancer were dominant, with a total of 9 studies [43,46-53]. A total of 2 studies identified were for lung cancer [54,55]. Of those targeting multiple tumor types, 1 recruited participants with breast or prostate cancer [45], and 1 recruited participants with lung, breast, or colorectal cancer [44]. The remaining 8 studies included several tumor types, such as participants living with any type or stage of cancer [42,56-58] or those with solid tumors attending ambulatory oncology clinics for chemotherapy [59,60].

#### Intervention Design and Features

The duration of interventions ranged from 4 weeks to 12 months, with a variable frequency of clinician-patient interactions ranging from biweekly to every 3 months. There were 5 studies involving the use of a web-based portal or web-based experience [45,46,48,55,57]; 8 studies included the use of a telephone or smartphone [43,44,47,52-54,59,60]; 2 studies used a combination of web-based and telephone interactions [51,58]; 2 studies used social media networks or social networks [51,55]; and 4 studies used wearable activity trackers [42,51-53]. Multidisciplinary care was identified in 30% (6/20) of the publications [44,46,47,55,56,58]. The study by Børøsund et al [46] was nurse-led, with referrals to either physicians or social workers. Uni-disciplinary interactions dominated, with 5 nurse-led [45,48,50,56,60], 1 social worker-led [49], and 1 led with a medical specialist experienced in mindfulness program delivery [42]. Bruggeman-Everts et al [41] involved a psychologist or physiotherapist assigned to participants in different arms of the study. Steel et al [57] outlined a collaborative care intervention, whereby a care coordinator provided information to the patient’s medical team, as well as patients and caregivers. The interventions included digital health tutoring, psychotherapy, nursing support, remote exercise or rehabilitation program delivery, and digital mindfulness.

Digital supportive care interventions included interactive voice response, tele and video counseling, internet-based patient-provider communication, exercise based on the internet, support systems, symptom monitoring, and self-management, mobile phone-based remote monitoring, and activity monitoring with tracking devices. The programs included digital health tutoring, psychotherapy, nursing support, remote exercise, rehabilitation program delivery, and digital mindfulness interventions. All varied in terms of design, features, and use of multimedia components. Only 2 publications referred to the CONSORT Digital Health Checklist [46,55].

### RoB Assessment

**Figure 2:**
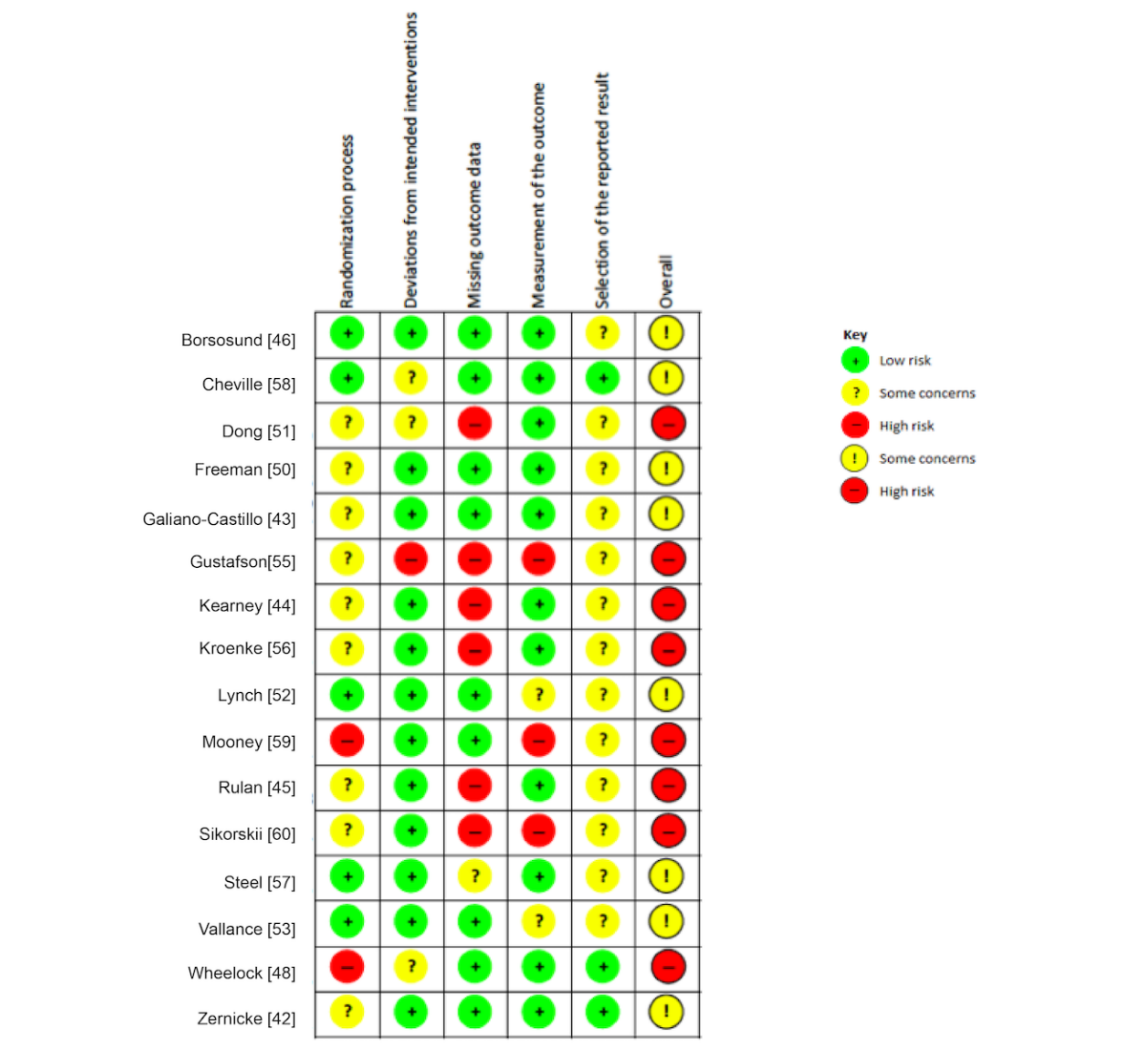
Risk of bias in studies with intention-to-treat analysis.

**Figure 3:**
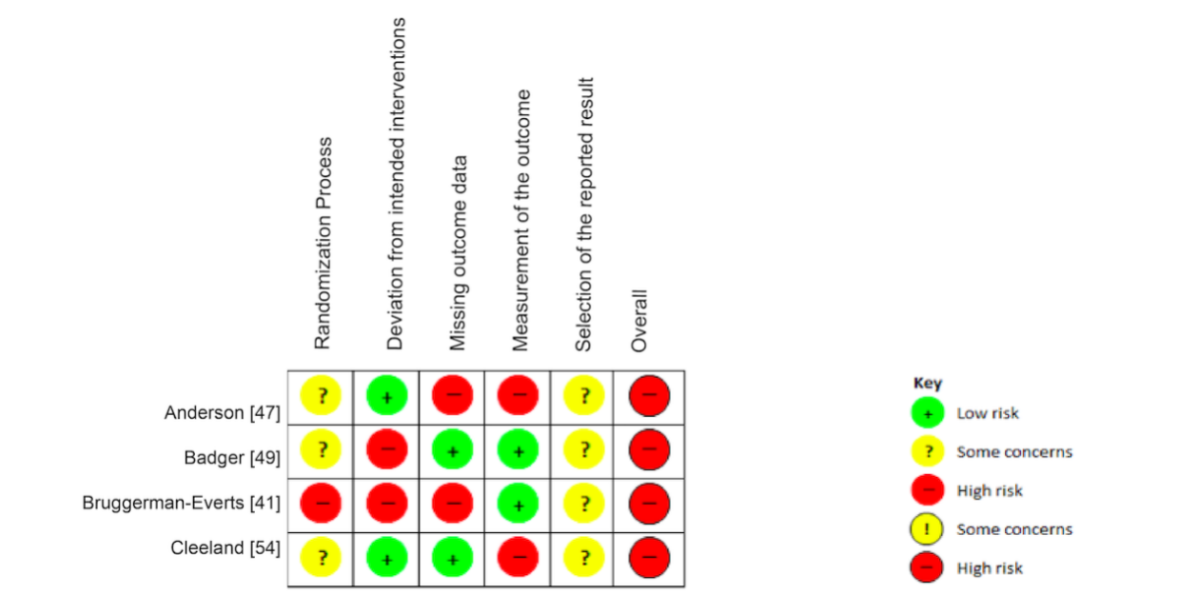
Risk of bias in studies with per-protocol analysis.

With regard to the overall RoB, no included studies were rated as *low* overall RoB; instead, 8 had *some concerns*, and 12 were *high risk*. A summary of the RoB assessment can be found in Multimedia Appendix 3 [41-60], describing the methodological quality of each domain according to the Cochrane tool for assessing RoB in randomized trials (RoB 2.0). Figures 2 and 3 describe a graphical representation of the RoB assessments. Figure 2 includes studies in which an intention-to-treat analysis was performed, whereas Figure 3 contains studies with a per-protocol analysis.

### Intervention Outcomes

#### Overview

Interventions were analyzed according to the before-after test design, with most of the interventions including repeated measurement points. Although 20 studies were included, the interventions and outcomes were heterogeneous and did not enable meta-analyses. Outcomes were synthetized using a model for quality of life among cancer survivors developed by Ferrell and Dow [61,62]. The model encompasses dimensions of physical, psychological, social, and spiritual well-being, specifying the content for each dimension in the context of cancer survivors. Details of the intervention outcomes and statistically significant results are presented in Table 1.

**Table 1:** Intervention outcomes.

Author	Primary and secondary outcomes (grouped)	Measures	Results
Anderson et al [47]	Pain Sleep Fatigue	MDASI^a^	Decreased pain severity from baseline to time point 1 (0.6 vs 2.3; *P*=.03; 95% CI 0.13 to 3.3) and from baseline to time point 2 (1.2 vs 3.5; *P*=.02; 95% CI 0.47 to 4.2) in the intervention group. Improved reported sleep.
Badger et al [49]	Depression Symptom distress Social well-being; spiritual well-being	CES-D^b^; GSDS^c^; Social and spiritual; Well-being scales	Depression, symptoms, and spiritual well-being improved in intervention groups (*P*=.01). No between-group differences. Social well-being improved for tele and video groups.
Børøsund et al [46]	Symptom distress Anxiety Depression Self-efficacy	MSAS^d^ HADS^e^ CBI^f^	WebChoice lower symptom distress (−0.16, 95% CI −.25 to −0.06; *P*=.001), anxiety (−0.79, 95% CI −1.49 to −0.09; *P*=.03), and depression (−0.79, 95% CI 1.18 to −0.05; *P*=.03) compared with control. Internet-based communication group lower depression (−0.69, 95% CI −1.32 to −0.05; *P*=.03) compared with usual care. No change in symptom distress or anxiety.
Bruggeman-Everts et al [41]	Fatigue severity Mental health Distress	CIS-FS^g^ The positive and negative affect schedule HADS	Clinically changes in fatigue severity in 66% (41/62) of patients in home-based physiotherapist guided protocol (AAF^h^), 49% (27/55) of patients in web-based psychologist-guided intervention (eMBCT^i^), and 12% (6/50) of patients in psycho-education e-mails only.
Cheville et al [58]	Functional capacity Pain HRQoL^j^	AM-PAC^k^ BPI^l^ 5-item EQ-5D-3L	Telerehabilitation by physical therapist-physician team (intervention group 1) improved function (difference, 1.3; 95% CI 0.08 to 2.35; *P*=.03) and quality of life (difference, 0.04; 95% CI 0.004 to 0.071; *P*=.01) compared with control. Intervention groups 1 and 2 showed reduced pain interference and average intensity (intervention group 1, −0.4; 95% CI −0.78 to −0.07; *P*=.02; and intervention group 2, −0.5; 95% CI −0.84 to −0.11; *P*=.006).
Cleeland et al [54]	Symptom threshold events Cumulative distribution of symptom threshold events Symptom severity	MDASI	Both groups had decreased symptom threshold events, control group reported more events at the end of the study period. For both groups together, the effect size of reduction in symptom severity was 0.72, effect size of 0.68 in the control group and 0.75 in the intervention group.
Dong et al [51]	HRQoL Muscle strength Cardiorespiratory capacity	SF-36^m^ Stand-up or sit-down chair test and arm lifting test (30 seconds) Modified Bruce treadmill protocol	CEIBISMS^n^ intervention showed improvements after 12 weeks in role-physical (*P*=.009), general health (*P*=.02), mental health (*P*=.01), vitality (*P*=.01) and health transition (*P*=.007). In comparison with control group, differences in vitality (*P*=.009), mental health (*P*=.001), and health transition (*P*=.048).
Freeman et al [50]	HRQoL Functional capacity Fatigue, sleep Spiritual well-being	SF-36 FACT-B^o^ FACIT-F^p^ and cog FACT-Sp^q^	Less fatigue (*P*=.002), cognitive dysfunction (*P*=.001), and sleep disturbance (*P*<.001) for both intervention groups compared with control. No differences between live delivery and telemedicine delivery of therapy. No group effect on overall quality of life; however, there was a time effect.
Galiano-Castillo et al [43]	Functional capacity Cognitive function	6-minute walk test Trail making test ACT^r^	After intervention, the telerehabilitation group had significantly improved distances as well as percentage of predicted 6-minute walk test compared with the control group (*P*<.001).
Gustafson et al [55]	Caregiver surveys reporting patient symptom distress	Modified ESAS^s^	Caregivers in the CHESS^t^ arm consistently reported lower patient physical symptom distress than caregivers in the internet arm at 4 months (*P*=.03); and at 6 months (*P*=.004)
Kearney et al [44]	6 chemotherapy-related symptoms	Common toxicity criteria adverse events chemotherapy symptom assessment scale	Difference between groups in fatigue, higher in the control group (OR^u^ 2.29, 95% CI 1.04 to 5.05; *P*=.04) and in hand-foot syndrome lower in control group (OR control or intervention 0.39, 95% CI 0.17 to 0.92; *P*=.03)
Kroenke et al [56]	Depression Pain severity	HSCL-20^v^ BPI	Improvements for the intervention group: >30% decrease in pain index (*P*<.001) and >50% decrease in the depression scale (*P*<.001). Effect size between-group differences at 3 months was 0.67 (95% CI 0.33 to 1.02) for pain and 0.42 (95% CI 0.16 to 0.69) for depression. Intervention group had better outcomes for several HrQoL domains, including mental health, vitality, anxiety, and physical symptom burden.
Lynch [52]^w^	Vigorous physical activity (MVPA^x^)	Actigraph and activPAL accelerometers	Between-group difference in MVPA at T2 (69 min/week; 95% CI 22 to 116); decreased total time of sitting 37 min/day (95% CI −72 to −2) and prolonged bouts of sitting 42 min/day (95% CI −83 to −2), favoring the intervention group
Vallance [53]^w^	HRQoL Fatigue	FACT-B FACIT-F	Intervention group improvement in fatigue at T2 4.6 (95% CI 1.3 to 7.8). Within groups: intervention group, increase in fatigue at T2 5.1 (95% CI 2.0 to 8.2) and at T-3 3.3 (95% CI 0.1 to 6.41). No effects on HRQoL.
Mooney et al [59]	Symptom severity Distress	Single item scale	No significant difference between symptom severity or distress scores between groups.
Ruland et al [45]	Symptom distress Depression Self-efficacy HRQoL	MSAS-SF^y^ Centre for Epidemiological Cancer Behavior Inventory Studies-Depression Scale 15 d	Decreased distress on one subscale of MSAS. Group differences on symptom distress were significant for the MSAS-SF (slope estimate, −0.052, 95% CI −0.101 to −0.004; t244=4.42; *P*=.04). There were no significant within- or between-group differences on the other MSAS-SF subscales.
Sikorskii et al [60]	Symptom severity	MDASI	Decreased symptom severity across both intervention groups after 10 weeks. No between-group differences. Effect sizes were similar for NASM^z^ (0.56) and ATSM^aa^ (0.59)
Steel et al [57]	Depression Pain Serum cytokine levels natural killer cell numbers	CES-D BPI Functional assessment of cancer therapy–anemia, and hepatobiliary	Reductions in pain (Cohen d=0.62), fatigue (Cohen d=0.26), depression (Cohen d=0.71), and significant changes in HRQoL with an effect size of Cohen d=0.99 at 6 months follow-up (*P*=.05) when compared with those in the enhanced usual care arm at 6 months.
Wheelock et al [48]	Time between symptoms Health care use	Clinic visits Health service use	Did not meet primary objective, no difference in health service use
Zernicke et al [42]	Feasibility Mood Stress Posttraumatic growth inventory	Monitoring interest, eligibility, and participation Profile of mood states CSOSI^ab^	Significant improvements and moderate effect sizes in the web-based MBCR^ac^ group relative to controls for mood disturbance (Cohen d=0.44; *P*=.049), stress (Cohen d=0.49; *P*=.02), spirituality (Cohen d=0.37; *P*=.04), and mindfully acting with awareness (Cohen d=0.50; *P*=.03).

^a^MDASI: MD Anderson symptom inventory.

^b^CES-D: Center for Epidemiological Studies-Depression.

^c^GSDS: General Sleep Disturbance Scale.

^d^MSAS: Memorial Symptom Assessment Scale.

^e^HADS: Hospital Anxiety And Depression Scale.

^f^CBI: Cancer Behavioral Inventory.

^g^CIS-FS: Checklist Individual Strength-Fatigue Severity.

^h^AAF: Ambulant Activity Feedback

^i^eMBCT: Web-based Mindfulness-Based Cognitive Therapy.

^j^HRQoL: health-related quality of life.

^k^AM-PAC: Activity Measure for Postacute Care.

^l^BPI: Brief Pain Inventory.

^m^SF-36: 36-item Short Form Health Survey.

^n^CEIBISMS: combined exercise intervention based on internet and social media software.

^o^FACT-B: Functional Assessment of Cancer Therapy–Breast.

^p^FACIT-F: Functional Assessment of Chronic Illness Therapy–Fatigue.

^q^FACT-Sp: Functional Assessment of Chronic Illness Therapy-Spiritual Well-Being.

^r^ACT: acceptance and commitment therapy.

^s^ESAS: Edmonton Symptom Assessment Score.

^t^CHESS: Comprehensive Health Enhancement Support System.

^u^OR: odds ratio.

^v^HSCL-20: Hopkins Symptom Checklist Depression Scale.

^w^Lynch [52] and Vallance [53] are 2 publications with different outcomes of the same randomized controlled trial.

^x^MVPA: moderate-to-vigorous intensity physical activity.

^y^MSAS-SF: Memorial Symptom Assessment Scale – Short Form

^z^NASM: nurse-assisted symptom management

^aa^ATSM: automated telephone symptom management

^ab^CSOSI: Calgary Symptoms of Stress Inventory.

^ac^MBCR: mindfulness-based cancer recovery.

#### Physical Well-being

Despite measuring similar outcomes, heterogeneous self-reported instruments were used across the studies. Control or reduction of symptoms and maintenance of function and independence comprise this domain. A total of 16 studies reported statistically significant changes in outcomes within the physical well-being domain [41,43-47,49,50,52-58,60]. Furthermore, 4 studies showed a significant reduction in pain [47,56-58], of which 3 used the Brief Pain Inventory [56-58]. Decreased fatigue was reported in 4 studies, favoring the intervention groups [41,44,50,53]. The most common outcome measure reported was clustered symptom changes, as referenced in 6 studies [45,46,49,54,55,60]. Finally, functional capacity-related outcomes were significant in 3 studies [43,52,58].

#### Health-Related Quality of Life

Health-related quality of life was the second most common domain, with significant improvements for the intervention group referenced in 4 studies [51,56-58].

#### Psychological Well-being

The most common outcome in this domain reporting significant improvements in the 4 interventions was depression [46,49,56,57]. Anxiety was reduced compared with the control group in one study [46], and significant improvements in mood disturbance were observed in one study [42].

#### Social and Spiritual Well-being

A single study reported a significant improvement in social well-being in the intervention group [49]. Badger et al [49] was also the only study that evaluated spiritual well-being changes, resulting in statistically significant improvements for the intervention group but no between-group differences.

## Discussion

### Principal Findings

#### Overview

In the studies integrated in this review, the interventions included digital health education, psychotherapy, nursing support, remote exercise, rehabilitation program delivery, and digital mindfulness interventions. All interventions satisfied the domains *health in our hands* and *interacting for health* of the digital health model by Shaw et al [13]. Digital supportive care interventions have been shown to improve cancer-related symptoms [45,46,49,54,55,60], pain [47,56-58], fatigue [41,44,50,53], health-related quality of life [51,56-58], functional capacity [43,52,58], and depression [46,49,56,57]. Only 2 RCTs included in this review did not report significant changes in one or more outcomes [48,59].

#### Digital Supportive Cancer Care Interventions

The digital interventions reviewed have been shown to be beneficial and independent of disease and demographic factors. This is similar to findings reported in other reviews [20,31,32,35,47,63] and meta-analyses [34]. In addition, the use of technology for cancer follow-up appears to be acceptable to patients, is clinically safe [31], and improves health knowledge and self-management practices [64]. However, such interventions vary in design and features, most lack or fail to report theoretical frameworks, and they use outcome measures making pooling or comparison between studies difficult. Another issue when comparing digital supportive care interventions is that it may be possible that interventions vary in their efficacy across different populations and technologies used for delivery. Furthermore, past studies have reported potential challenges impacting the implementation of digital health care, such as technical problems, lack of technology knowledge, and data security [65], which need to be considered when planning future studies. Recent systematic reviews concluded that a range of strategies should be implemented in digital supportive care [20,35,63] and general digital health interventions [66]. The study by O’Connor et al [66] recommends increasing public awareness of different technologies and understanding of how they work, personalization of care, clinical accreditation of interventions, improving focus on health literacy, and safeguarding privacy of personal information as key areas for investigation. Key areas for digital supportive care design and implementation noted in previous cancer-related reviews that should be further explored are mechanisms for participant feedback to drive the co-design of digital interventions [20,67], the efficiency of delivering relevant and tailored health care information [65], and ways to integrate supportive care services at all stages of the cancer treatment pathway [35].

A supportive digital care intervention model should be underpinned by a theoretical framework that anticipates not only the outcomes and the tools to measure these, but also the process of achieving the outcomes from a particular intervention [20,63,68]. However, many studies do not address the validity of patient-reported health outcomes, and most of them use self-reported measures in pre-post test design, which leaves them at a RoB. This might be in part because one of the main challenges in the development of an evidence-based digital supportive cancer care intervention is the velocity of technology development in comparison with the often-long process of conducting and evaluating clinical trials.

#### Digital Health for Chronic Disease Care

Evidence from the research and implementation of digital health interventions across other disease groups may facilitate the transferability of digitally enabled supportive cancer care. Cancer is a chronic disease for many people [69]. Applications of research findings in other chronic diseases, such as cardiovascular disease [70], hypertension [12], and diabetes [71], which have a larger evidence base in digital health–enabled interventions with positive effects, should be used where possible. A recent systematic review [72] focused on the broader application of these symptom-reporting systems within multiple patient groups and concluded that although further research needs to be completed, most studies reported positive health outcomes. For example, in the case of diabetes, Greenwood et al [71] found that the most effective digital interventions incorporated all components of a technology-assisted self-management feedback loop, connected people with diabetes and their health care team using two-way communication, analyzed patient-generated health data, tailored education programs, and individualized feedback. A 2018 systematic review focused on using remote monitoring in people with a history of type 2 diabetes [73] significantly improved glycated hemoglobin and self-management. Evidence from diabetes research seems more cohesive, in part, because the outcomes of lowering glucose levels and glycated hemoglobin allow homogeneous measurement across studies.

Several successful digital health interventions focus on both behavior change and increasing patient engagement [74,75]. Barello [75] concluded that most studies failed to account for the complexity of patient engagement and that a more holistic approach might help maximize the potential of digital health technology [75]. Another recent review focused on mobile health apps for chronic disease management and found that regular symptom assessments, automated reminders, and feedback loops were common features, with most studies reporting significant improvement in health outcomes [73].

### Current Challenges and Need for Quality Information

This review compiles evidence regarding the potential of digital health interventions for supportive cancer care in different settings, including remote areas and emergency situations [33]. However, the challenges facing public health systems worldwide in terms of emergencies, such as the COVID-19 pandemic, have rapidly increased the use of digital health interventions [29]. Health care systems, including cancer care, are adapting in response to the need for social distancing, lockdowns, and other public health initiatives. Cancer clinics have reduced clinical appointments, administration encounters, and postponed elective cancer surgeries [28], which has increased the need for follow-up and management without visiting hospitals [76]. This situation has advanced the use of digital health and telehealth apps and programs worldwide [30]. Digital supportive cancer care has been implemented out of necessity and is becoming a common delivery model [77]. Two of the suggested strategies to enable supportive cancer care during COVID-19 are (1) empowering patients and caregivers through the use of digital communication and (2) increasing the use of existing digital health platforms [28].

There is an urgent need to agree on relevant outcomes, methods of assessment, and there is a need for improved quality of primary studies and RCTs, as shown by our quality assessment. The lack of high-quality randomized trials identified in this review reflects the ongoing problem of low-quality research. Moreover, only 2 of the included publications referenced the CONSORT Digital Health Checklist, which was published in 2011. Only 3 included studies were published before 2011, making it disappointing that most later publications failed to reference this standard. Comments of Dickinson in BMC Cancer [31] remain relevant in 2020:

Nevertheless, there are surprisingly few randomized trials given the explosion in technological innovation in recent years. It could be that technology is evolving so fast that potential innovative technological interventions become outdated before they can mature sufficiently to be subjected to randomized trials.

This insight has been recurrent in different systematic reviews [32,74].

### Strengths and Limitations

Although the review was a rigorous evaluation of RCTs, there were a small number of included studies that indicated there may be significant literature in the phase I or II feasibility spectrum. Although 20 studies were included, the interventions and outcomes were heterogeneous and did not enable meta-analyses. Owing to the nature of this review, there was also a heterogeneous population, variable outcome measures, variable study quality, and methodological limitations. These characteristics have been found to contribute to a lack of evidence regarding the benefits of digital health [19]. It is difficult to draw conclusions and synthesize studies with inconsistent outcome measures, and a systematic approach to using standardized measures is required. The CONSORT Digital Health Checklist should be used routinely. The review was limited to studies written in English. Therefore, it is possible that research papers published in other parts of the world were missed. As with many studies in oncology, this review found that breast cancer survivorship dominated. Thus, the development of supportive care interventions across other tumor streams is required. As most of the included studies were conducted in the United States and Europe, it is unclear whether the findings from these studies can be generalized to other countries and populations, particularly in developing nations.

### Future Directions

Living well with cancer has gained greater relevance as the survival rates of many cancer types are increasing. The future of digital health in oncology supportive care brings a range of new and exciting possibilities. There is a need to evaluate the efficacy and efficiency of digital interventions in real-world conditions and standardize a core set of outcomes included in all studies to facilitate comparisons between interventions and digital technologies.

### Conclusions

Digital health–enabled supportive cancer care is capable of improving health-related quality of life, symptom burden including self-report of pain and fatigue, depression, and, to a lesser extent, functional capacity. Supportive digital interventions in the field of cancer are being used and have been reported to be helpful for patients, independent of other factors. However, there is a need for higher quality research and clearer reporting than is evident in the current RCTs. Future research should focus on using valid, standardized outcome measures, increasing the methodological rigor of studies undertaken, and the development and evaluation of strategies to improve both patient and health professional engagement in the design and delivery of supportive digital health interventions.

## Appendix

Multimedia Appendix 1 Search strategy.

Multimedia Appendix 2 Population and intervention characteristics.

Multimedia Appendix 3 Summary of risk of bias assessment.

## Abbreviations

CONSORT: Consolidated Standards of Reporting Trials
PRISMA: Preferred Reporting Items for Systematic Reviews and Meta-Analyses
PROSPERO: International Prospective Register of Systematic Reviews
RCT: randomized controlled trial
RoB: risk of bias
