# Supplementary material for: Supportive Care Interventions for People With Cancer Assisted by Digital Technology: Systematic Review
Source: J Med Internet Res. 2021 Oct 29;23(10):e24722. doi: 10.2196/24722 (PMC8590193; doi:10.2196/24722)
Supplement: Multimedia Appendix 1 [file jmir_v23i10e24722_app1.docx]

## Multimedia Appendix: Search Strategy

| (( supportive care ) OR ( allied health ) OR ( delivery of health care ) OR ( physical activity ) OR ( rehab* ) OR ( model of care ) OR ( patient education ) OR ( patient support ) ) AND ( ( ehealth OR digital health OR mhealth OR telehealth OR telerehabilitation OR webbased OR apps OR wearables OR devices OR information comunication technology ) ) AND ( cancer patient* OR oncology patient* OR cancer survivor* AND PUBYEAR > 2000 AND ( LIMIT-TO ( LANGUAGE , "English" ) ) |
| --- |
