# Supplementary material for: Supportive Care Interventions for People With Cancer Assisted by Digital Technology: Systematic Review
Source: J Med Internet Res. 2021 Oct 29;23(10):e24722. doi: 10.2196/24722 (PMC8590193; doi:10.2196/24722)
Supplement: Multimedia Appendix 2 [file jmir_v23i10e24722_app2.docx]

## Multimedia Appendix – Population and Intervention Characteristics

| Author, location | Sample size | Population | Intervention | Control | Health professional interaction(s) | Intervention detail(s) and time points |
| --- | --- | --- | --- | --- | --- | --- |
| Anderson et al [47]; United States | - n=60 - Intervention=31 - Control=29 | African American and Latina women with breast cancer | Management system automated, telephone-based, IVR^a^ | Usual care | Alerts over threshold sent to medical oncologists for review | - Automated system called patient twice weekly for 8 weeks. - Baseline measures to all patients during clinic visits at time point 1 (4-6 weeks after enrollment) and at time point 2 (8-10 weeks after enrollment). |
| Badger et al [49]; United States | - n=52 - Intervention 1=18 - Intervention 2=20 - Intervention 3=14 | Women with breast cancer and partner dyad | - Intervention 1: Tele-education - Intervention 2: Telecounseling - Intervention 3: Video counseling | No control group | - Carer-patient - Social worker-patient - Social worker-carer | - Eight weekly sessions, partners 4 sessions fortnightly delivered individually. - All participants were assessed 3 times: baseline following recruitment (T1), at 8-week intervention (T2), and final survey (T-3) 8 week after the T2 survey. |
| Borosund et al [46]; Norway | - n=167 - Intervention 1=64 - Intervention 2=45 - Control=58 | Women with breast cancer | - Intervention 1: WebChoice, an interactive web-based health support system and e-messages (IPPC^b^) - Intervention 2: IPPC services/e-messages only | Usual care | - Intervention 1: Nurse-led, with referral to physician and social worker and patient-patient (forum) - Intervention 2: Nurse-led, with referral to physician and social worker | Consenting patients completed baseline questionnaires before randomization. Then, at 2, 4, and 6 months after enrolling. |
| Bruggeman-Everts et al [41]; Netherlands | - n=167 - Intervention 1=62 - Intervention 2=55 - Control=50 | Cancer patients with a history of cancer-related fatigue | - Intervention 1: Home-based physiotherapist guided protocol (AAF^c^) - Intervention 2: Web-based psychologist-guided intervention (eMBCT^d^) | Psychoeducational e-mails only | - Intervention 1: Physiotherapist-patient - Intervention 2: psychologist-patient | 9 weeks. Participants had finished curative-intent cancer treatment at least 3 months previously and had been suffering from severe fatigue ever since (≥35 on the CIS-FS^e^ subscale). |
| Cheville et al [58]; United States | - n=516 - Intervention 1=172 - Intervention 2=172 - Control=172 | Patients stage IIIC or IV of cancer with moderate functional impairment | - Intervention 1: Telerehabilitation by physical therapist-physician team - Intervention 2: Telerehabilitation plus pharmacological pain management by nurse | Automated monitoring for pain and usual care | - Intervention 1: Physical therapist and physician team-patient - Intervention 2: Additional nurse-patient | - 6 months of centralized telerehabilitation. - Baseline assessment before randomization, then 3 and 6 months by blinded telephone interviewer. |
| Cleeland et al [54]; United States | - n=100 - Intervention=50 - Control=50 | Patients with lung cancer | Automated symptom monitoring coupled with email feedback to clinicians | Automated monitoring and usual symptom care | Alerts sent to cancer specialist on the basis of set thresholds for intervention group. | Symptoms reported twice weekly for 4 weeks. Baseline measure before discharge from the hospital (approximately 5 days after surgery). Patients completed a paper-and-pencil version of the MDASI^f^ (T0). |
| Dong et al [51]; China | - n=60 - Intervention=30 - Control=30 | Women with breast cancer phase I to III | Exercise intervention on the basis of internet and social media software (CEIBISMS^g^) including phone, step-recording app; face-to-face remote video instruction, social media apps | Usual care | Physiotherapists—patient | 12 weeks CEIBISMS^g^. Physical rehabilitation training televideo face-to-face, 3 times/week 30-min sessions. Cardiorespiratory capacity training via televideo face-to-face 4 times/week. Postoperative cancer-related therapy within 4 months to 2 years. Assessments at baseline and 12 weeks after intervention. |
| Freeman et al [50]; United States | - n=118 - Intervention 1=48 - Intervention 2=23 - Control=47 | Women with breast cancer | - Intervention 1: Live delivery, Imagery-based behavioral intervention - Intervention 2: Telemedicine delivery; Imagery-based behavioral intervention | Waitlist | - Nurse-patient - Intervention 2: Additional face-to-face contact with other consumers | 5 weekly 4-hour sessions delivered to group of 15-20 participants either face-to-face or remotely plus short weekly telephone call (<10 min) for 12 weeks. 7 self-report measures of QOL^h^ were examined at baseline (before randomization), 1, and 3-months post-treatments. |
| Galiano-Castillo et al [43]; Spain | - n=81 - Intervention=40 - Control=41 | Women with breast cancer | Internet-based tailored exercise program (CUIDATE^i^), includes instant messaging, video conferencing, telephone calls | Usual care | Research staff-patient | 8 weeks, a total of 24 sessions were included in the exercise program (3 sessions per week) with a duration of 90 min/day. Assessments at baseline, after intervention (8 weeks), and follow-up at 6 months after intervention. |
| Gustafson et al [55]; United States | - n=285 - Intervention=144 - Control=141 | Lung cancer patients and caregiver dyad | eSupport system (CHESS^j^) information, and support (peers, clinicians, experts, and social networks) | Usual care plus internet information | - Caregiver–patient dyad-clinician - Caregiver–patient dyad peers | - 25 months’ access or 13 months after patient’s death. CHESS^j^ access 24/7. - Patients with perceived life expectancy of at least 4 months. |
| Kearney et al [44]  United Kingdom | - n=112 - Intervention=56 - Control=56 | Lung, breast, and colorectal patients on chemotherapy | Mobile phone-based, remote monitoring, ASyMS^k^ | Usual care | Automated through mobile phone. Intervention group alerts to nurse and physician. | Symptoms reported on days 1-14 over 4 cycles of chemotherapy. All patients completed a paper version of questionnaire at their prechemotherapy assessment and before chemotherapy cycles 2, 3, 4, and 5. |
| Kroenke et al [56]  United States | - n=405 - Intervention=202 - Control=203 | Cancer patients with a history of depression, pain, or both | Centralized telecare management by a nurse-physician specialist team coupled with automated home-based symptom monitoring by interactive voice recording or internet | Usual care | - Automated survey - Phone calls: Nurse-patient (plus nurse—pain psychiatrist specialist) | Baseline before randomization and 3 follow-up calls (1, 4, and 12 weeks), scheduled telephone contacts, with additional triggered telephone calls based on alerts. |
| Lynch et al [52] Australia; Vallance et al [53] Australia | - n=83 - Intervention=43 - Control=40 | Postmenopausal women with stage 1-3 breast cancer after primary treatment | ACTIVATE: Activity Monitor coupled with behavioral feedback, goal-setting session and 5 telephone-delivered health coaching sessions. | Waitlist | ACTIVATE Trial team member-patient | - 12-week period. - Activity assessments at baseline (T1) and at the end of the intervention (T2). - HRQoL^m^ and fatigue additional follow-up (T-3) at 24 weeks. |
| Mooney et al [59]; United States | - n=250 - Intervention=129 - Control=121 | Patients with cancer receiving chemotherapy | Automated information technology–based telephone symptom monitoring system, plus email alerts report on preset thresholds | Automated information technology–based telephone symptom monitoring system only | Symptoms exceeding preset thresholds for moderate-to-severe intensity generated email alert reports to both the patient’s oncologist and oncology nurse. | Patients were screened after their first cycle of chemotherapy. Commenced 24 h after chemotherapy, beginning with cycle 2, and continuing through cycle 3. An average of 45 observed days per participant. |
| Ruland et al [45]  Norway | - n=325 - Intervention=162 - Control=163 | Breast and prostate cancer patients | WebChoice (internet-based, symptom monitoring, tailored information and self-management support, e-communication with clinicians, and peer forum) | Links to cancer-related websites | Nurse-patient, plus Patient-patient (Platform to share their experiences with other patients and obtain professional oncology nursing support). | 1-year follow-up. Symptom distress was measured 5 times: At baseline before randomization and at 3, 6, 9, and 12 months. |
| Sikorskii et al [60]; United States | - n=437 - Intervention 1=219 - Intervention 2=218 | Men and women with cancer | - Intervention 1: ATSM^n^ - Intervention 2: NASM^o^ delivered by telephone by cancer nurses | Patients scoring <2 on symptoms were called twice weekly for 6 weeks | - Intervention grp 1: Automated - Intervention grp 2: Nurse | - 6 contacts, 8-week ATSM^n^ intervention delivered via an automated system. - Patients were screened for symptom severity, randomized, then at 10 weeks, outcome data were obtained through second interview. |
| Steel et al [57]; United States | - n=261 - Intervention=144 - Control=117 | Men and women with advanced cancer and family caregivers | Web-based, stepped collaborative care intervention | Enhanced usual care | - Care coordinator (trained in CBT^p^)-patient - Phone calls and face-to-face clinic Consult care coordinator provides information to patient’s medical team. Family also enrolled in study. | Six-month intervention Telephone contact with the care coordinator approximately every 2 weeks and face-to-face contact approximately every 2 months. Interviews and blood draws were performed 7 weeks after the cancer-related treatment to avoid capturing treatment-related side effects. |
| Wheelock et al [48]; United States | - n=102 - Intervention=59 - Control =41 | Women with breast cancer | SIS.NET^q^(System for Individualized Survivorship Care) remote nurse monitoring, included free text fields for patient questions and generated automated referrals with predefined thresholds | Usual care | Nurse-patient (Nurse monitored web-based questionnaires and provided telephone follow-up) | 18-month intervention. Baseline symptoms documented at the time of study enrollment before randomization. 3 clinic visits. Web-based health questionnaires at 3-month intervals between clinic visits. |
| Zernicke et al [42]; Canada | - n=62 - Intervention=30 - Control=32 | Men and women with cancer | Synchronous web-based MBCR^r^ | Waitlist | Teacher-patient, participants were able to see, hear, and interact in real time with other group members and the instructor during the web-based synchronous intervention. | - Weekly 2-hour sessions for 8 weeks as well as opportunity for extended practice. Web-based 6-hour retreat between weeks 6 and 7 of the course. - 3 years after primary cancer treatment for inclusion. All completed pre-(T1), post-(T2) wait intervention assessment; and (T-3) post-MBCR^r^ assessment. |

^a^IVR: interactive voice response.

^b^IPPC: internet-based patient-provider communication.

^c^AAF: Ambulant Activity Feedback

^d^eMBCT: Web-based mindfulness-based cognitive therapy.

^e^CIS-FS: Checklist Individual Strength - Fatigue Severity.

^f^MDASI: The MD Anderson Symptom Inventory.

^g^CEIBISMS: combined exercise intervention based on internet and social media software.

^h^QoL: quality of life.

^i^CUIDATE: e-CUIDATE telehealth system.

^j^CHESS: Comprehensive Health Enhancement Support System.

^k^ASyMS: Advanced Symptom Management System.

^l^Lynch and Vallance (2019) are 2 publications with different outcomes of the same RCT.

^m^HRQoL: health-related quality of life.

^n^ATSM: automated telephone symptom management.

^o^NASM: nurse-assisted symptom management.

^p^CBT: cognitive behavioral therapy

^q^SIS.NET: System for Individualized Survivorship Care

^r^MBCR: mindfulness-based cancer recovery.
