# Supplementary material for: Supportive Care Interventions for People With Cancer Assisted by Digital Technology: Systematic Review
Source: J Med Internet Res. 2021 Oct 29;23(10):e24722. doi: 10.2196/24722 (PMC8590193; doi:10.2196/24722)
Supplement: Multimedia Appendix 3 [file jmir_v23i10e24722_app3.docx]

## Multimedia Appendix – Summary of risk of bias assessment

| Reference | Domain 1: Randomisation Process | Domain 2: Deviations from intended Intervention | Domain 3: Missing Outcome Data | Domain 4: Measurement of the Outcome | Domain 5:  Selection of the Reported Results | Overall  Risk of Bias |
| --- | --- | --- | --- | --- | --- | --- |
|  |  |  |  |  |  |  |
| Anderson 2015 | *Some Concerns* Insufficient randomization details, no information about allocation concealment. | *Low* | *High* Attrition was higher in the intervention group, no sensitivity analysis was performed to assess the effect of missing data. | *High* Intervention characteristics could have influenced outcome reporting. | *Some Concerns* Insufficient detail on pre-specified analysis plans. | *High* |
| Badger 2013 | *Some Concerns* Insufficient randomization details, no allocation concealment. | *High*  No patient or provider blinding.  Intervention technical difficulties may have affected outcomes. No ITT analysis. | *Low* | *Low* | *Some Concerns* Insufficient details on pre-specified analysis plans. | *High* |
| Borosund 2014 | *Low* | *Low* | *Low* | *Low* | *Some Concerns* Insufficient details on pre-specified analysis plans.  Deviation from initial sample size calculation | *Some Concerns* |
| Bruggerman -Everts 2017 | *High* Impaired randomization process, unclear details on allocation concealment, group differences at baseline. | *High* Intervention characteristics could have influenced the outcome reporting. | *High* Different dropout rates with no sensitivity analysis conducted. | *Low* | *Some Concerns* Insufficient details on pre-specified analysis plans. | *High* |
| Cheville  2019 | *Low* | *Some Concerns*  No patient blinding. No information about potential deviations. | *Low* | *Low* | *Low* | *Some*  *Concerns* |
| Cleeland 2011 | *Some Concerns* No details on allocation concealment. | *Low* | *Low* | *High* Intervention characteristics could have influenced outcome reporting. | *Some Concerns* Insufficient details on pre-specified analysis plans. | *High* |
| Dong 2019 | *Some Concerns*  Baseline characteristics differ between groups. | *Some Concerns*  No patient or provider blinding. | *High*  Different dropout rates with no sensitivity analysis conducted. | *Low* | *Some Concerns*  Insufficient details on pre-specified analysis plans. | *High* |
| Freeman 2015 | *Some Concerns* No details on allocation concealment. | *Low* | *Low* | *Low* | *Some Concerns* Insufficient details on pre-specified analysis plans. | *Some Concerns* |
| Galiano-Castillo 2017 | *Some Concerns* Groups were similar at baseline except for the menopause variable. | *Low* | *Low* | *Low* | *Some Concerns* Insufficient details on pre-specified analysis plans. | *Some Concerns* |
| Gustafson 2013 | *Some Concerns* No details on allocation concealment. | *High* Difficult to compare proportion of participants who deviated because the authors did not track 'use' in the control group. | *High* Possible relationship between outcomes and their missingness. | *High* Inappropriate outcome measurement. | *Some Concerns* Insufficient details on pre-specified analysis plans. | *High* |
| Kearney 2009 | *Some Concerns* No details on allocation concealment. | *Low* | *High* Possible relationship between outcomes and missingness. | *Low* | *Some Concerns* Insufficient details on pre-specified analysis plans. | *High* |
| Kroenke 2010 | *Some Concerns* No details on allocation concealment. | *Low* | *High* Possible relationship between outcomes and missingness. | *Low* | *Some Concerns* Insufficient details on pre-specified analysis plans. | *High* |
| Lynch 2019 | *Low* | *Low* | *Low* | *Some Concerns*  No patient or provider blinding.  Intervention characteristics could have influenced outcome measurements. | *Some Concerns*  It wasn't defined what cutpoints would be consider to report results. | *Some Concerns* |
| Mooney 2014 | *High* Insufficient randomization details, no allocation concealment. | *Low* | *Low* | *High* Possible differences in outcome reporting between groups. | *Some Concerns* Insufficient details on pre-specified analysis plans. | *High* |
| Ruland 2013 | *Some Concerns* No details on allocation concealment. | *Low* | *High* Missing outcomes with no sensitivity analysis. | *Low* | *Some Concerns* Insufficient details on pre-specified analysis plans. | *High* |
| Sikorskii 2007 | *Some Concerns* No details on allocation concealment. | *Low* | *High* There's a high likelihood that missingness of outcomes was related to their true values. | *High* Inconsistent outcome measurements. | *Some Concerns* Insufficient details on pre-specified analysis plans. | *High* |
| Steel 2016 | *Low* | *Low* | *Some Concerns* High attrition due to death, but rates were fairly similar between groups. Many patients who remained in the intervention didn’t complete outcome measures. | *Low* | *Some Concerns* Insufficient details on pre-specified analysis plans. | *Some Concerns* |
| Vallance 2019 | *Low* | *Low* | *Low* | *Some Concerns*  No patient or provider blinding.  Intervention characteristics could have influenced outcome measurements. | *Some Concerns*  Insufficient details on pre-specified analysis plans. | *Some Concerns* |
| Wheelock 2015 | *High* Unclear randomization details, no allocation concealment and unequal distribution of confounders at baseline. | *Some Concerns* The electronic interface may have affected participants' ability to complete questionnaires. | *Low* | *Low* | *Low* | *High* |
| Zernicke 2014 | *Some Concerns* Uneven group distribution of cancer type and disease stage at baseline. | *Low* | *Low* | *Low* | *Low* | *Some Concerns* |
